# What Is the Explanation?

**Published:** 1891-04

**Authors:** G. H. Ellis

**Affiliations:** Maxwell, Iowa


### What Is the Explanation?

The following special notice came to hand from Iowa a few days ago. It is a "*rare bit.*" Such statements in regard to prices as is here given always bear evidence of deception, and a total lack of the "*esprit de corps*" that is an inherent quality of every truly professional man.

The aim and effort of every one should be to enlarge the sphere of his usefulness by extending his experience, increasing his skill, and serving those who come into his charge better and better as day by day he goes on in his professional career. The dentist who puts his skill, professional ability, and aspirations on a mercantile—barter—level with corn, potatoes, and poultry certainly is not entitled to much rank as a professional man—indeed, he does not deserve any rank at all. No one who voluntarily depreciates, or lowers the value of his service, is likely to make progress—on the other hand he is sure to retrograde. This will occur in spite of any resolution he may make to the contrary.

"I will give three years warrantee on all classes of work."

What folly. As well might the physician give a guarantee on the health of his patient. Such promises are always indicative of gross ignorance, or fraud. Note further: "I have had most fourteen years of heavy practice since leaving the Dental

College at Ann Arbor, Mich., and feel competent that I can render you honest work."

Can any thing be more incongruous than this talk about honesty when the facts are that the statement in the quotation just given is absolutely false in every aspect? This person is not a graduate of the dental college at Ann Arbor, was never a member of its freshman class, and even so far as is known, never made a visit to the college.

Now Mr. G. H. Ellis (D.D.S.?) if you have any reasonable explanation to make of the statement contained in this card, send it on, and none will be more gratified than the writer of this criticism, to make correction, and if the explanation is a good vindication it shall have as wide a circulation as this may have, but put it in good English. It is not often that one man gets a whole page of advertising in the REGISTER free, but we always feel so affable and pleasant after receiving such cards (and they come frequently) that the impulse is to do something for somebody—some kind act, or give some good advice. Don't do it again; stick to the truth if it kills you; but it won't.

But lest the reader wearies here is the card:

DENTISTRY.

As it has been most two years since I located in Maxwell, and the public have so liberally patronized me, I thought in as much as prices in farm produce were so low, I would for this summer put my prices as follows: Full upper and lower set of teeth on rubber base, $16.00. Either upper or lower set on silver, $12.00, on gold, $25.00. Filling, enameling, 50 cents. Gold and Platinum, 75 cents. Malgam, 50 cents. All Gold $1.25, to $2.00. Extracting reasonable. I will give three years warrantee on all classes of work. I have had most fourteen years of heavy practice since leaving the Dental college at Ann Harbor, Mich., and feel competent that I can render you honest work.

G. H. Ellis, D.D.S.

Maxwell, Iowa.

